# 5,6-Dimethoxybenzofuran-3-one derivatives: a novel series of dual Acetylcholinesterase/Butyrylcholinesterase inhibitors bearing benzyl pyridinium moiety

**DOI:** 10.1186/2008-2231-21-15

**Published:** 2013-02-27

**Authors:** Hamid Nadri, Morteza Pirali-Hamedani, Alireza Moradi, Amirhossein Sakhteman, Alireza Vahidi, Vahid Sheibani, Ali Asadipour, Nouraddin Hosseinzadeh, Mohammad Abdollahi, Abbas Shafiee, Alireza Foroumadi

**Affiliations:** 1 Department of Medicinal Chemistry, Faculty of Pharmacy and Neurobiomedical Research Center, Shahid Sadoughi University of Medical Sciences, Yazd, 8915173143, Iran; 2 Faculty of Pharmacy and Pharmaceutical Sciences Research Center, Tehran University of Medical Sciences, Tehran, Iran; 3 Herbal Medicine Center, Shahid Sadoughi University of Medical Sciences, Yazd, 8915173143, Iran; 4 Neuroscience Research Center, Kerman University of Medical Sciences, Kerman, Iran; 5 Drug design & Development Research Center, Tehran University of Medical Sciences, Tehran, Iran

## Abstract

**Background:**

Several studies have been focused on design and synthesis of multi-target anti Alzheimer compounds. Utilizing of the dual Acetylcholinesterase/Butyrylcholinesterase inhibitors has gained more interest to treat the Alzheimer’s disease. As a part of a research program to find a novel drug for treating Alzheimer disease, we have previously reported 6-alkoxybenzofuranone derivatives as potent acetylcholinesterase inhibitors. In continuation of our work, we would like to report the synthesis of 5,6-dimethoxy benzofuranone derivatives bearing a benzyl pyridinium moiety as dual Acetylcholinesterase/Butyrylcholinesterase inhibitors.

**Methods:**

The synthesis of target compounds was carried out using a conventional method. Bayer-Villiger oxidation of 3,4-dimethoxybenzaldehyde furnished 3,4-dimethoxyphenol. The reaction of 3,4-dimethoxyphenol with chloroacetonitrile followed by treatment with HCl solution and then ring closure yielded the 5,6-dimethoxy benzofuranone. Condensation of the later compound with pyridine-4-carboxaldehyde and subsequent reaction with different benzyl halides afforded target compounds. The biological activity was measured using standard Ellman’s method. Docking studies were performed to get better insight into interaction of compounds with receptor.

**Results:**

The in vitro anti acetylcholinesterase/butyrylcholinesterase activity of compounds revealed that, all of the target compounds have good inhibitory activity against both Acetylcholinesterase/Butyrylcholinesterase enzymes in which compound 5b (IC50 = 52 ± 6.38nM) was the most active compound against acetylcholinesterase. The same binding mode and interactions were observed for the reference drug donepezil and compound 5b in docking study.

**Conclusions:**

In this study, we presented a new series of benzofuranone-based derivatives having pyridinium moiety as potent dual acting Acetylcholinesterase/Butyrylcholinesterase inhibitors.

## Introduction

Alzheimer’s disease (AD) is a progressive and age-dependent neurodegenerative brain disorder that leads to dementia, cognitive impairment, and memory loss [1,2]. The main cause of Alzheimer’s disease etiology is not completely known however, many diverse factors such as hippocampal acetylcholine (Ach) decrease, β-amyloid (Aβ) aggregation and τ-protein deposits seem to play significant roles in initiation and progression of the disease [3-5]. Based on these findings three hypotheses was established: cholinergic, β-amyloid and tau hypothesis.

**Figure 1:**
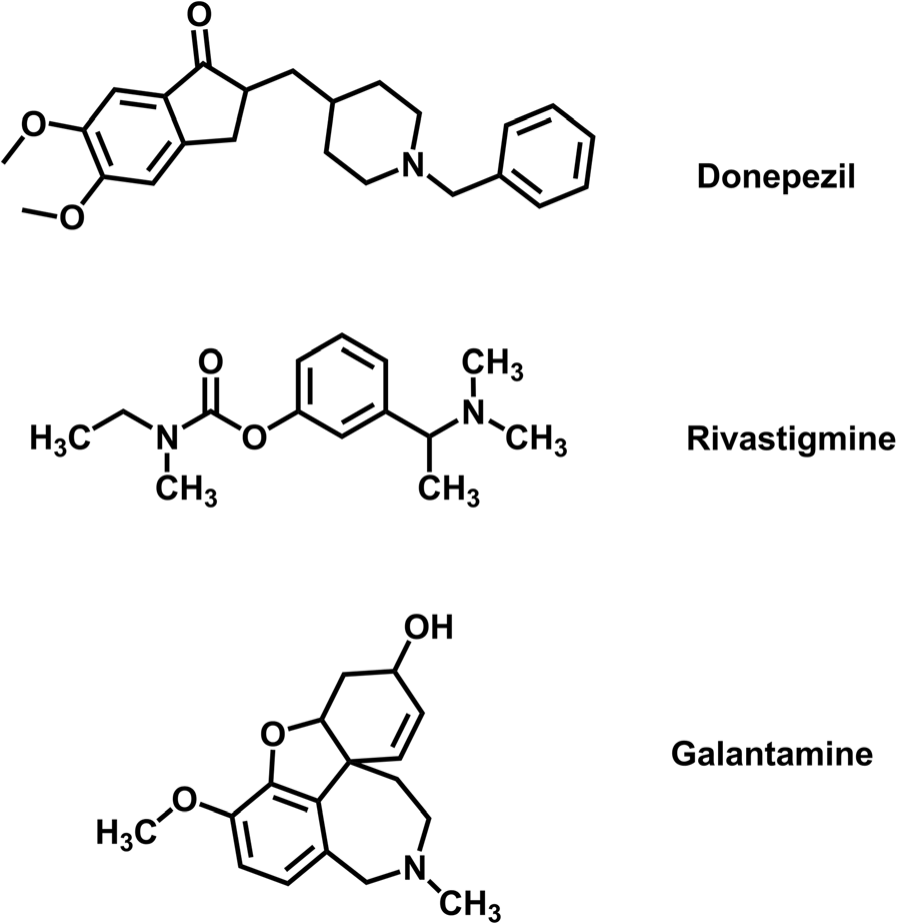
Donepezil, Rivastigmine and Galantamine three well-known AChE inhibitors.

Regarding cholinergic hypothesis one of the most useful approaches for improving AD’s symptoms is to design new agents that raise the acetylcholine in the cholinergic system [6]. Acetylcholinesterase (AChE) is responsible for hydrolysis of acetylcholine in the synaptic cleft, therefore; employing the AChE inhibitors could be a helpful strategy to increase the level of acetylcholine in the damaged cholinergic neurons [7]. Several compounds have been previously synthesized as AChE inhibitors and successfully used to treat AD, such as Donepezil, Galantamine and Rivastigmine (Figure 1) [8].

Moreover, it has been demonstrated that the inhibition of AChE may lead to an increase in Butyrylcholinesterase (BuChE) activity in the hippocampus that causes hydrolysis of AChE by a new way. Therefore, the maintenance of AChE/BuChE activity ratio in the hippocampus as seen in the healthy brain, could improve the signs and symptoms of AD [9]. As a result, design and synthesis of dual AChE/BuChE inhibitors should be considered to find more potent agents against AD [10].

**Figure 2:**
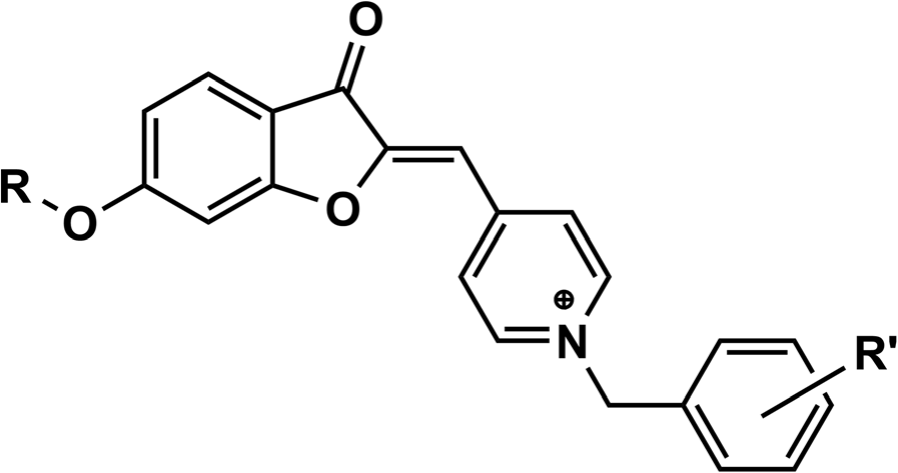
**Previously reported (*Z*)-1-benzyl-4-((6-alkoxy-3-oxobenzofuran-2(3*H*)-ylidene) methyl)pyridinium derivatives as potent inhibitors of AChE.**

In an earlier report, we have presented the preparation and evaluation of some new (*Z*)-1-benzyl-4-((6-alkoxy-3-oxobenzofuran-2(3*H*)-ylidene) methyl)pyridinium (Figure 2) as AChE inhibitors [11]. Most of these compounds proved to be potent AChE inhibitors in vitro, among which compounds bearing methoxy group on position 6 of benzofuran ring showed the most activity. Furthermore, it was reported that 5,6-dimethoxy benzofuranone structure is important to show more affinity toward the enzyme in some aurone-based AChE inhibitors [12,13]. Following these reasons and in pursuit of our previous study a series of new 5,6-dimethoxy benzofuran derivatives were designed, synthesized and evaluated for AChE/BuChE inhibitory activities. In this study, we decided to investigate the possible increase in the enzyme inhibitory activity by replacing the less hydrophilic 6-alkoxy benzofuranone scaffold with a more polar 5,6-dimethoxy benzofuranone motif. The final compounds have been tested for their ability to inhibit both AChE and BuChE using Ellman’s method [14].

## Materials and methods

### Chemistry

**Figure 3:**
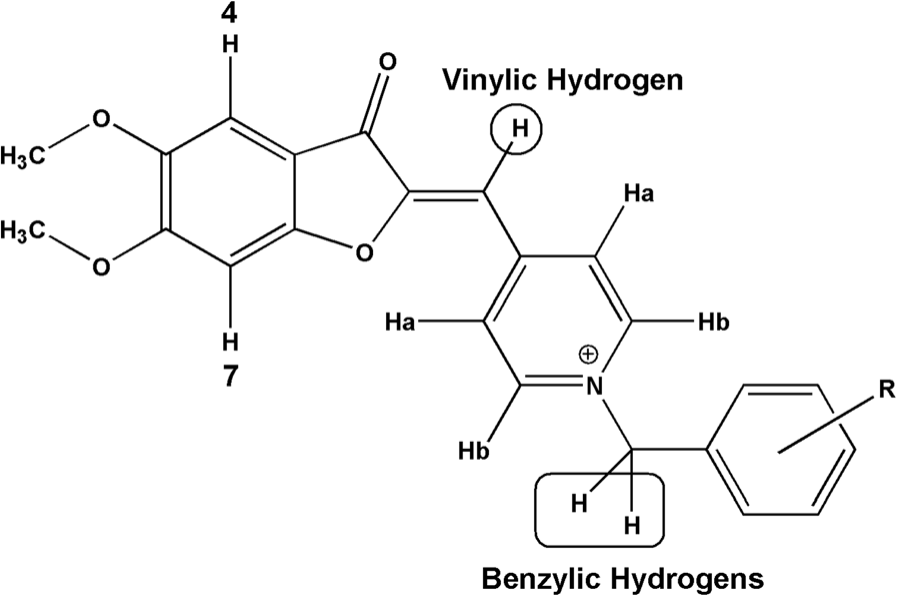
General structure and atom numbering of final compounds.

All chemicals were obtained from Merck AG, Aldrich and Acros Chemicals. Thin layer chromatography (TLC) was carried out on Merck pre-coated silica gel F254 plates with various mobile phase systems, to check reaction progress and product mixtures. The Separating column chromatography and flash chromatography were done with silica gel (70-230 mesh). The ^1^H nuclear magnetic resonance (NMR) spectra were recorded in DMSO-d_6_ and/or CDCl_3_ on a Bruker FT-500 MHz spectrometer with tetramethylsilane (TMS) as the internal standard. Coupling constants were reported in Hertz (Hz) and chemical shifts are given as δ value (ppm) relative to TMS as internal standard. To express spin multiplicities, s (singlet), d (doublet), t (triplet), q (quartet), dd (double doublet) and m (multiplet) were used. Mass spectra were obtained at 70 eV in a Finigan TSQ-70 spectrometer. Infrared (IR) spectra were determined using a Nicolet FT-IR Magna 550 spectrophotometer. All melting points were determined using Kofler hot stage apparatus and are uncorrected.Elemental microanalyses were done on a Perkin–Elmer 240-C apparatus for C, H, and N. For better understanding of spectral data, the general structure and atom numbering of final compounds are depicted in Figure 3.

#### Synthesis of 3,4-dimethoxyphenol (1)

A mixture of *m*-chloro-peroxybenzoic acid (*m*-CPBA) (7 g, 32.5 mmol) in the dry dichloromethane (DCM) (40 ml) was prepared at 0°C and vigorously stirred. Then a solution of 3,4-dimethoxybenzaldehyde (5 g, 30 mmol) in the dry DCM (10 ml) was added dropwise during 1 hour. The resulting mixture was kept at room temperature and then refluxed for 16 hours. After cooling, the mixture was washed with aqueous solution of saturated sodium hydrogen carbonate (3 × 20 ml) followed by washing with sodium thiosulfate 10% (25 ml) to neutralize excess amount of *m*-CPBA. The solvent was then evaporated under reduced pressure and the resulting crude material was dissolved in methanol. The solution was stirred for 4 hours with excess amount of sodium hydroxide 10% solution at room temperature. The pH of solution was adjusted to 1 using HCl 6 N solution. The solution was then extracted with DCM (3 × 25 ml) washed with brine and dried using anhydrous Na_2_SO_4_. The solvent was removed to yield brown syrup, which was purified with column chromatography using petroleum ether and ethyl acetate (1:1) as eluent to give compound 1. White brown solid, m.p. 74–78°C, 84% yield [15].

#### Synthesis of 2-chloro-1-(2-hydroxy-4,5-dimethoxyphenyl)ethanone (2)

To a mixture of 3,4-dimethoxyphenol (1) (4.93 g, 32 mmol) and chloroacetonitrile (2.4 g, 32 mmol) in dry ether (150 ml) was added anhydrous ZnCl_2_ (1.44 g, 10.6 mmol). The mixture was cooled to 0°C and HCl gas was bubbled through the reaction for 2.5 hours. The mixture was left in the room temperature overnight and then cooled to 0°C. The precipitated iminium was filtered off and washed three times with ether. The imine was dissolved in 160 ml of 1 N HCl and refluxed for 90 min. The resulting mixture was extracted with DCM (3 × 100 ml) and the solvent was removed under reduced pressure to give 2.88 g white brown solid 40% yield (no further purification was needed).

#### Synthesis of 5,6-dimethoxybenzofuran-3(2H)-one (3)

The crude extract of 2-chloro-1-(2-hydroxy-4,5-dime-thoxyphenyl)ethanone (2) (1.15 g, 5 mmol) was dissolved in 10 ml ethanol and then refluxed for 10 min under argon. Sodium acetate trihydrate (700 mg) was added thereto and refluxed for 10 min again. The resulting mixture was cooled immediately and then filtered off. The solvent was evaporated and compound 3 was yielded by re-crystallization from ethanol. White needle crystals, m.p. 163–165, 470 mg, 48%. IR ν_max_/cm^-1^ (KBr) : 1708 (C = O), ^1^H NMR (CDCl_3_, 80 MHz), 7.02 (s, 1H, H_4-aromatic_) 6.59 (s, 1H, H_7-aromatic_) 4.61 (s, 2H, H_2-aliphatic_) 3.97 (s, 3H, -OCH_3_) 3.87 (s, 3H, -OCH_3_), EI-MS *m/z* (%) 194 (M^+^, 100), 165 (45), 135 (88), 34 (57).

#### Synthesis of (Z)-5,6-dimethoxy-2-(pyridin-4-ylmethylene)benzofuran-3(2H)-one (4)

5,6-dimethoxybenzofuran-3(*2H*)-one 3 (388 mg, 2 mmol), pyridine-4-carbaldehyde (308 mg, 2.88 mmol) and PTSA (548 mg, 3.18 mmol) were suspended in the dry toluene (25 ml) and refluxed using Dean-Stark apparatus for 2 hours. After cooling the resulting mixture, the precipitated solid was filtered off. The wet solid was suspended in aqueous 10% NaHCO_3_ solution (50 ml) and stirred for 60 minutes at room temperature. The filtered resulting solid was washed with water (50 ml) and then air dried. The crude solid was purified by re-crystallization from acetonitrile to yield compound 4. 400 mg, 70% yield, m.p. 265–268°C, IR ν_max_/cm^-1^ (KBr) : 1705 (C = O), 1635 (C = C alkene), ^1^H NMR (CDCl_3_, 80 MHz), 8.69 (dd, 2H, H_a-pyridine_, *J* = 4.5 Hz, *J* = 2 Hz), 7.70 (dd, 2H, H_b-pyridine_, *J* = 5 Hz, *J* = 1.5 Hz), 7.17 (s, 1H, H_4_), 6.83 (s, 1H, H_7_), 6.69 (s, 1H, H_vinylic_), 4.03 (s, 3H, -OCH_3_), 3.91 (s, 3H, -OCH_3_), EI-MS *m/z* (%) 284 ( (M^+^ + 1), 100), 224 (90), 33 (65).

#### General procedure for the synthesis of 1-benzyl-4-((5,6-dimethoxy-3-oxobenzofuran-2(3H)-ylidene)methyl) pyridinium halide derivatives (5a-g)

(*Z*)-5,6-Dimethoxy-2-(pyridin-4-ylmethylene) benzofuran-3(2*H*)-one (4) (1 equiv.), was suspended in 7 ml dry acetonitrile and heated under reflux condition. Afterwards different substituted benzyl halides (1.2 equiv.) were added thereto. The mixture was refluxed for 2–3 hours followed by cooling to room temperature. After that the solvent was evaporated and 15 ml n-hexane was added to the residue. The resulting mixture was filtered off and the precipitated crystals were separated, washed with n-hexane and dried. Flash chromatography of the crystals using the chloroform-methanol (99–1) as the mobile phase, furnished the final compounds 5a-g.

#### (Z)-1-Benzyl-4-((5,6-dimethoxy-3-oxobenzofuran-2(3H)-ylidene) methyl) pyridinium bromide (5a)

Starting from (*Z*)-5,6-dimethoxy-2-(pyridin-4-ylmethylene)benzofuran-3(2*H*)-one (1 mmol, 0.283 g) and benzyl bromide (1.2 mmol, 0.205 g), compound 5a was obtained, quantitative yield, mp over 300°C, IR ν_max_/cm^-1^ (KBr) : 1689 (C = O), 1607 (C = C alkene), ^1^H NMR (DMSO-d_6_, 500 MHz), 9.20 (d, 2H, H_a-Pyridine_, *J =* 6.7 Hz), 8.51 (d, 2H, H_b-pyridine_, *J =* 6.7 Hz), 7.55-7.46 (m, 5H, H_phenyl_), 7.27 (s, 1H, H_4-benzofuranone_), 7.25 (s, 1H, H_7-benzofuranone_), 7.04 (s, 1H, H_vinylic_), 5.85 (s, 2H, H_benzylic_), 3.98 (s, 3H, -OCH_3_), 3.83(s, 3H, -OCH_3_), EI-MS *m/z* (%) 283 (10), 91 (100), 77 (15), 43 (38), Anal. Calcd.for C_23_H_20_BrNO_4_: C, 60.81; H, 4.44; N, 3.08 Found: C, 60.68; H, 4.72; N, 3.24.

#### (Z)-1-(4-Fluorobenzyl)-4-((5,6-dimethoxy-3-oxobenzofuran-2(3H)-ylidene) methyl) pyridinium bromide (5b)

Starting from (*Z*)-5,6-dimethoxy-2-(pyridin-4-ylmethylene)benzofuran-3(2*H*)-one (1 mmol, 0.283 g) and 4-fluoro-benzyl bromide (1.2 mmol, 0.226 g), compound 5b was obtained, quantitative yield, mp 276–279°C, IR ν_max_/cm^-1^ (KBr) : 1687 (C = O), 1608 (C = C alkene), ^1^H NMR (DMSO-d_6_, 500 MHz), 9.22 (d, 2H, H_a-pyridine_, *J =* 6.7 Hz), 8.52 (d, 2H, H_b-pyridine_, *J =* 6.75 Hz), 7.67-7.64 (m, 2H, H_phenyl_), 7.32-7.28 (m, 2H, H_phenyl_), 7.25 (s, 1H, H_4-benzofuranone_), 7.23 (s, 1H, H_7-benzofuranone_), 7.04 (s, 1H, H_vinylic_), 5.86 (s, 2H, H_benzylic_), 3.97 (s, 3H, -OCH_3_), 3.82(s, 3H, -OCH_3_), EI-MS *m/z* (%) 283 (12), 109 (100), 95 (18), 43 (40), Anal. Calcd.for C_23_H_19_BrFNO_4_: C, 58.49; H, 4.05; N, 2.97 Found: C, 58.85; H, 4.17; N, 2.74.

#### (Z)-1-(3-Methylbenzyl)-4-((5,6-dimethoxy-3-oxobenzofuran-2(3H)-ylidene) methyl) pyridinium chloride (5c)

Starting from (*Z*)-5,6-dimethoxy-2-(pyridin-4-ylmethylene)benzofuran-3(2*H*)-one (1 mmol, 0.283 g) and 3-methyl-benzyl chloride (1.2 mmol, 0.169 g), compound 5c was obtained, quantitative yield, mp 293–295°C, IR ν_max_/cm^-1^ (KBr) : 1694 (C = O), 1605 (C = C alkene), ^1^H NMR (DMSO-d_6_, 500 MHz), 9.25 (d, 2H, H_a-pyridine_, *J =* 6.4 Hz), 8.51 (d, 2H, H_b-pyridine_, *J =* 6.45 Hz), 7.48-7.41 (m, 3H, H_phenyl_), 7.33 (s, 1H, H_phenyl_), 7.26 (s, 1H, H_4-benzofuranone_), 7.24 (s, 1H, H_7-benzofuranone_), 7.03 (s, 1H, H_vinylic_), 5.83 (s, 2H, H_benzylic_), 3.94 (s, 3H, –OCH_3_), 3.80 (s, 3H, –OCH_3_), 2.3 (s, 3H, H –CH_3phenyl_), EI-MS *m/z* (%) 283 (12), 105 (100), 91 (16), 43 (38), Anal. Calcd.for C_24_H_22_ClNO_4_: C, 68.00; H, 5.23; N, 3.30 Found: C, 68.34; H, 5.05; N, 3.42.

#### (Z)-1-(2-Fluorobenzyl)-4-((5,6-dimethoxy-3-oxobenzofuran-2(3H)-ylidene) methyl) pyridinium chloride (5d)

Starting from (*Z*)-5,6-dimethoxy-2-(pyridin-4-ylmethylene)benzofuran-3(2*H*)-one (1 mmol, 0.283 g) and 2-fluorobenzyl chloride (1.2 mmol, 0.173 g), compound 5d was obtained, quantitative yield, mp over 300°C, IR ν_max_/cm^-1^ (KBr) : 1701 (C = O), 1608 (C = C alkene), ^1^H NMR (DMSO-d_6_, 500 MHz), 9.19 (d, 2H, H_a-pyridine_, *J =* 5.8 Hz), 8.53 (d, 2H, H_b-pyridine_, *J =* 5.95 Hz), 7.65-7.62 (m, 1H, H_phenyl_), 7.54-7.52 (m, 1H, H_phenyl_), 7.34-7.31 (m, 2H, H_phenyl_), 7.26 (s, 1H, H_4-benzofuranone_), 7.23 (s, 1H, H_7-benzofuranone_), 7.05 (s, 1H, H_vinylic_), 5.94 (s, 2H, H_benzylic_), 3.95 (s, 3H, –OCH_3_), 3.80 (s, 3H, –OCH_3_), EI-MS *m/z* (%) 283 (16), 109 (100), 95 (18), 43 (35), Anal. Calcd.for C_24_H_22_ClNO_4_: C, 68.00; H, 5.23; N, 3.30 Found: C, 68.12; H, 5.32; N, 3.37.

#### (Z)-1-(3-Fluorobenzyl)-4-((5,6-dimethoxy-3-oxobenzofuran-2(3H)-ylidene) methyl) pyridinium chloride (5e)

Starting from (*Z*)-5,6-dimethoxy-2-(pyridin-4-ylmethylene) benzofuran-3(2*H*)-one (1 mmol, 0.283 g) and 3-fluorobenzyl chloride (1.2 mmol, 0.173 g), compound 5e was obtained, quantitative yield, mp 291–294°C, IR ν_max_/cm^-1^ (KBr) : 1690 (C = O), 1611 (C = C alkene), ^1^H NMR (DMSO-d_6_, 500 MHz), 9.29 (d, 2H, H_a-pyridine_, *J =* 6 Hz), 8.52 (d, 2H, H_b-pyridine_, *J =* 6.2 Hz), 7.51-7.50 (m, 2H, H_phenyl_), 7.43-7.41 (m, 1H, H_phenyl_), 7.30-7.27 (m, 1H, H_phenyl_), 7.26 (s, 1H, H_4-benzofuranone_), 7.23 (s, 1H, H_7-benzofuranone_), 7.04 (s, 1H, H_vinylic_), 5.9 (s, 2H, H_benzylic_), 3.95 (s, 3H, –OCH_3_), 3.8 (s, 3H, –OCH_3_), EI-MS *m/z* (%) EI-MS *m/z* (%) 283 (19), 109 (100), 95 (15), 43 (32), Anal.Calcd.for C_23_H_19_ClFNO_4_: C, 64.57; H, 4.48; N, 3.27 Found: C, 64.72; H, 4.23; N, 3.16.

#### (Z)-1-(2-Methylbenzyl)-4-((5,6-dimethoxy-3-oxobenzofuran-2(3H)-ylidene) methyl) pyridinium chloride (5f)

Starting from (*Z*)-5,6-dimethoxy-2-(pyridin-4-ylmethylene)benzofuran-3(*2H*)-one (1 mmol, 0.283 g) and 2-methyl-benzyl chloride (1.2 mmol, 0.169 g), compound 5f was obtained, quantitative yield, mp 287–290°C, IR ν_max_/cm^-1^ (KBr) : 1692 (C = O), 1607(C = C alkene), ^1^H NMR (DMSO-d_6_, 500 MHz), 9.08 (d, 2H, H_a-pyridine_, *J =* 6.85 Hz), 8.52 (d, 2H, H_b-pyridine_, *J =* 6.8 Hz), 7.81- 7.78 (m, 1H, H_phenyl_), 7.45-7.38 (m, 1H, H_phenyl_), 7.32-7.30 (m, 2H, H_phenyl_), 7.29 (s, 1H, H_4-benzofuranone_), 7.24 (s, 1H, H_7-benzofuranone_), 6.99 (s, 1H, H_vinylic_), 5.88 (s, 2H, H_benzylic_), 3.99 (s, 3H, –OCH_3_), 3.84 (s, 3H, –OCH_3_), 2.35 (s, 3H, H CH_3phenyl_) EI-MS *m/z* (%) 283 (21), 105 (100), 91 (18), 43 (40), Anal. Calcd.for C_24_H_22_ClNO_4_: C, 68.00; H, 5.23; N, 3.30 Found: C, 68.26; H, 5.15; N, 3.25.

#### (Z)-1-(4-Methylbenzyl)-4-((5,6-dimethoxy-3-oxobenzofuran-2(3H)-ylidene) methyl) pyridinium chloride (5g)

Starting from (*Z*)-5,6-dimethoxy-2-(pyridin-4-ylmethylene)benzofuran-3(*2H*)-one (1 mmol, 0.283 g) and 4-methylbenzyl chloride (1.2 mmol, 0.169 g), compound 5g was obtained, quantitative yield, mp 294–297°C, IR ν_max_/cm^-1^ (KBr) : 1690 (C = O), 1606 (C = C alkene), ^1^H NMR (DMSO-d_6_, 500 MHz), 9.24 (d, 2H, H_a-pyridine_, *J =* 6.4 Hz), 8.49 (d, 2H, H_b-pyridine_, *J =* 6.5 Hz), 7.47 (d, 2H, *J =* 6.3 Hz, H_phenyl_), 7.26 (d, 2H, *J =* 6.4 Hz, H_phenyl_), 7.20 (s, 1H, H_4-benzofuranone_), 7.17 (s, 1H, H_7-benzofuranone_), 6.96 (s, 1H, H_vinylic_), 5.80 (s, 2H, H_benzylic_), 3.95 (s, 3H, –OCH_3_), 380 (s, 3H, –OCH_3_), 2.28 (s, 3H, H –CH_3phenyl_), EI-MS *m/z* (%) 283 (17), 105 (100), 91 (22), 43 (31), Anal. Calcd.for C_24_H_22_ClNO_4_: C, 68.00; H, 5.23; N, 3.30 Found: C, 67.76; H, 5.38; N, 3.19.

### Biological activity

AChE (AChE, E.C. 3.1.1.7, Type V-S, lyophilized powder, from *electric eel,* 1000 unit), Cholinesterase from equine serum were purchased from Sigma–Aldrich (Steinheim, Germany). DTNB (5, 5^′^-Dithiobis-(2-nitrobenzoic acid)), KH_2_PO_4_, K_2_HPO_4_, KOH, NaHCO_3_, BTChI (butyrylthio-choline iodide) and ATChI (acetylthiocholine iodide) were obtained from Fluka (Buchs, Switzerland). To afford an assay concentration range (10^-4^ to 10^-9^ M), the tested compounds were dissolved in a mixture of 20 ml distilled water and 5 ml methanol followed by dilution in 0.1 M KH_2_PO_4_/K_2_HPO_4_ buffer (pH 8.0) to obtain final concentration.

The colorimetric Ellman’s method was applied to evaluate anti AChE/BuChE activity of tested compounds. The solutions temperature was adjusted to 25°C prior to use. Five different concentrations of each compound were tested to obtain 20% to 80% inhibition of AChE and/or BuChE activity. The assay medium contained 3 ml of 0.1 M phosphate buffer pH 8.0, 100 μl of 0.01 M 5, 5^′^-dithio-bis(2-nitrobenzoic acid), 100 μl of 2.5 unit/mL enzyme solution (AChE, E.C. 3.1.1.7, Type V-S, lyophilized powder, from *electric eel* or BuChE from equine serum).

Then 100 μl of each tested compounds, were added to the assay medium and pre-incubated at 25°C for 15 min followed by adding 20 μl of substrate (acetylthiocholine iodide or butyrylthiocholine iodide). After that the rate of absorbance change was measured at 412 nm for 2 minutes. The blank reading solution was used to justify non-enzymatic hydrolysis of substrate during the assay. The blank solution contained 3 ml buffer, 200 μl water, 100 μl DTNB and 20 μl substrate. As a reference, an identical solution of the enzyme without the inhibitor is processed following the same protocol. The rate of the substrate enzymatic hydrolysis was calculated, and % inhibition of the tested compounds was calculated. Each concentration was evaluated in triplicate, and the IC_50_ values were determined from inhibition curves (% of inhibition versus log inhibitor’s concentration) graphically. Kapková et al. reported more details of the procedure [16,17].

### Docking study

Docking simulation studies were done using Autodock Vina 1.1.1 [18]. For this purpose, the pdb structure of acetylcholinesterase (1EVE) was retrieved from the Brookhaven protein database (RCSB)(http://www.rcsb.org) as a complex bound with inhibitor Donepezil. Subsequently, all water molecules and the co-crystallized ligand were removed from the pdb structure. Afterward the polar hydrogens were added to the receptor and pdbqt format of the receptor was created using Autodock Tools 1.5.4 [19].The ligand coordinates were generated using Marvine-Sketch 5.8.3, 2012, ChemAxon (http://www.chemaxon.com). Then the structures were converted to pdbqt using Open babel 2.3.1 [20]. The docking site was defined by establishing a box at geometrical center of the native ligand present in the above mentioned PDB structure with the dimensions of 40, 40, and 40. The exhaustiveness parameter was set to 80 and the box center was set to the dimensions of x = 2.023, y = 63.295, z = 67.062. Finally, the lowest energy conformations between the AChE and inhibitor were selected for analyzing the interactions. The results were visualized using Chimera 1.6 [21].

## Results and discussion

### Chemistry

**Scheme 1:**
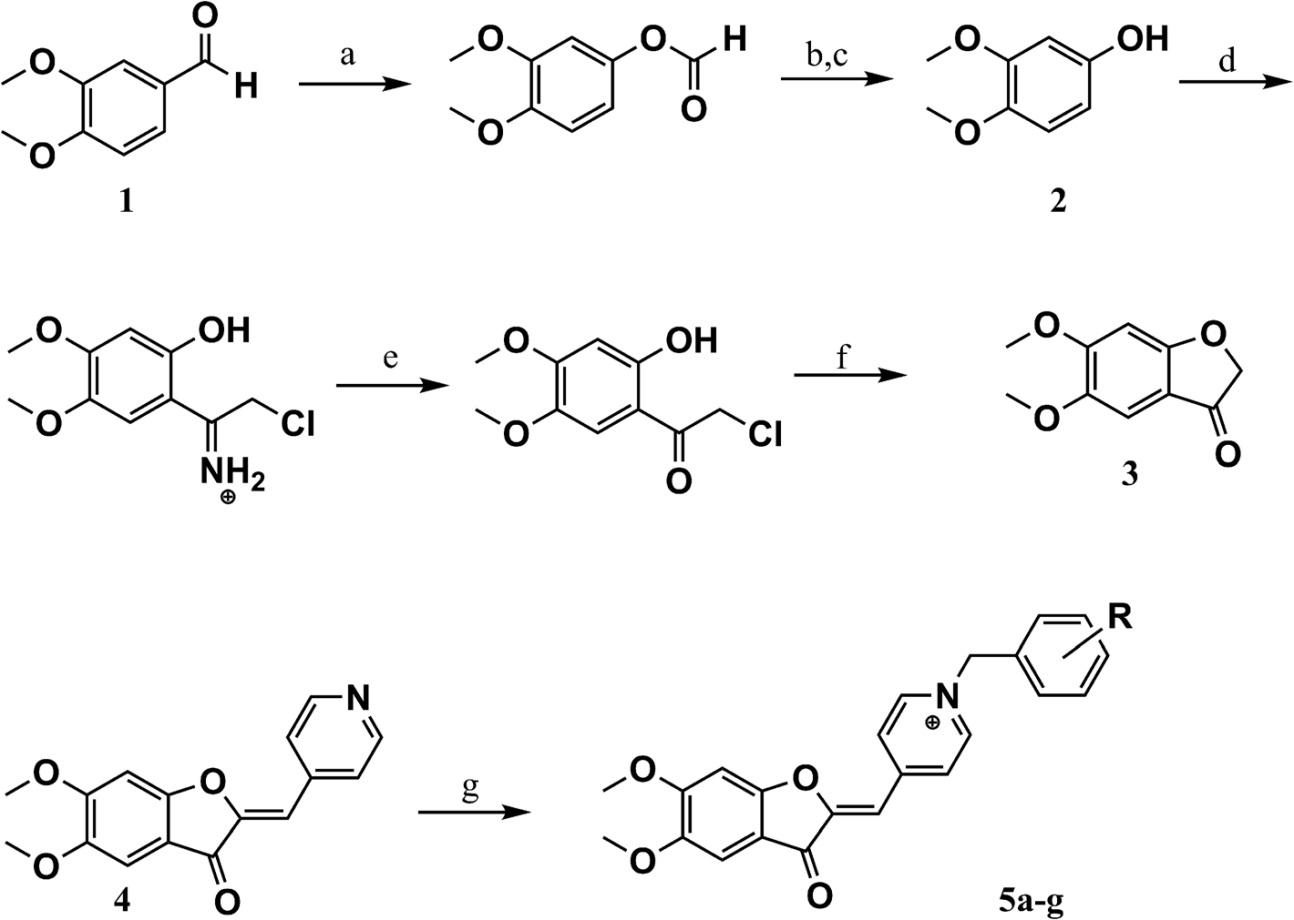
**Synthetic routes to final compounds 5a-g (a) *m*-CPBA, CH2Cl2, reflux 16 hrs; (b) NaOH 10**%**, r.t., stir., 4 hrs; (c) HCl 6 N; (d) ClCH2CN, HCl gas, ZnCl2, 0°C, 2.5 hrs, stir.; (e) HCl 1 N, reflux, 90 min; (f) Sodium acetate trihydrate, ethanol, reflux, 10 min; (g) Pyridine-4-carboxaldehyde, PTSA, toluene, reflux; (h) Substituted benzyl halide, CH3CN, reflux.**

The target compounds (5a-g) were synthesized as illus-trated in Scheme 1. The convenient Baeyer–Villiger oxidation of 3,4-dimethoxybenzaldehyde furnished 3,4-dimethoxyphenol (1) in a good yield [15]. Reaction of 3,4-dimethoxyphenol (1) with chloroacetonitrile using ZnCl_2_ as Lewis acid yielded 2-chloro-1-(2-hydroxy-4,5-dime-thoxyphenyl)ethane iminium as a key intermediate. Afterwards, 2-chloro-1-(2-hydroxy-4,5-dimethoxyphenyl)ethanone (2) was prepared followed by treatment of iminium intermediate using hydrochloric acid. Intermolecular cyclization of compound (2) using sodium acetate resulted in formation of 5,6-dimethoxy-benzofuran-3(2*H*)-one (3) [22]. Condensation of 5,6-dimethoxybenzofuran-3(2*H*)-one (3) with 4-formyl pyridine in the presence of PTSA gave (*Z*)-5,6-dimethoxy-2-(pyridin-4-ylmethylene)benzofuran-3(2*H*)-one (4). As previously reported, the configuration of exocyclic formed double bond was assigned as *Z* (*cis*) isomer [11]. Appropriate benzyl chloride or bromide derivatives were refluxed with compound (4) in dry acetonitrile to obtain final compounds (5a-g).

### Biological activity

The new series of 5,6-dimethoxybenzofuranone derivatives bearing the benzyl pyridinium moiety was evaluated for AChE/BuChE activity using slightly modified Ellman’s protocol.

The type and position of substituents on benzyl moiety could alter the electronic and steric properties of the phenyl ring. Therefore, the affinity of the target compounds toward the enzyme could be modified by changing the substituent on benzyl moiety.

The anti AChE/BuChE activity data of the target compounds were summarized in Table 1.

**Table 1:** **AChE/BuChE inhibitory activity of compounds 5a-g compared with Donepezil hydrochloride (IC**_**50**_ **= Mean ± SD)**

	
**Compounds**	**R**	**AChE (IC**_**50**_ **± SD)**^**a **^**(nM)**	**BuChE (IC**_**50**_ **± SD)**^**a **^**(nM)**	**Selectivity for AChE**^**b **^**(S.I)**

**5a**	H	86 ± 10.94	1400 ± 85	16.27
**5b**	2-F	52 ± 6.38	1620 ± 73	31.15
**5c**	3-F	115 ± 15.56	740 ± 23	6.43
**5d**	4-F	74 ± 11.32	960 ± 41	12.97
**5e**	2-CH_3_	262 ± 27.49	3620 ± 84	13.81
**5f**	3-CH_3_	208 ± 31.72	5310 ± 115	25.52
**5g**	4-CH_3_	514 ± 29.53	7600 ± 260	14.78
Donepezil hydrochloride	-	31 ± 5.12	5400 ± 95	174.19

^a ^Data are means ± standard deviation of three independent experiments.

^b ^Selectivity Index (S.I): IC_50_ BuChE/ IC_50_ AChE.

According to the data, all of the synthesized compounds have shown considerable anti AChE/BuChE activity however, did not reach the inhibition level of the reference compound (Donepezil hydrochloride IC_50_ = 31 ± 5.12 nM).

As already shown in our previous study, the substitution on phenyl ring with small electron withdrawing group such as fluorine atom makes the compounds more active rather than their unsubstituted counterparts. In an opposite way the methyl phenyl substituted compounds proved to be less active than the unsubstituted compounds. In this study using the identical substitution on the ring, the anti cholinesterase activity of 5,6-dimethoxy-benzofuranone derivatives has changed in the same way. Regardless of the substitution on phenyl ring, the activity of more polar 5,6-dimethoxybenzofuranone derivatives were slightly diminished, compared to 6-alkoxy benzofuranone derivatives that were previously reported [11].

As anticipated, the compound 5b (IC_50_ = 52 ± 6.38 nM) containing 2-F substituent exhibited the highest inhibitory activity toward the enzyme. However, its activity was less than its counterparts bearing 6-methoxy (IC_50_ = 10 ± 6.87 nM), 6-ethoxy (IC_50_ = 32 ± 7.75 nM) and 6-propoxy (IC_50_ = 50 ± 9.86) on benzofuranone.

Similarly, the 3 and 4-flouro phenyl compounds have shown lower activity in respect to corresponding 6-alkoxy benzofuranone series.

According to Table 1, changing the *ortho* position of fluorine to either *meta* or *para* decreased the activity as in compound (5c) with significantly diminished activity (IC_50_ = 115 ± 15.56).

Accordingly, methyl substituted compounds have exhibited the order of activity as follow: 5f > 5e > 5g. Insertion of methyl group on phenyl ring in any position reduced the activity in comparison with unsubstituted compound (5a).

In an accord to these findings, it was concluded that substitution on position 3 with F and position 4 with –CH_3_ was not tolerated.

### Docking simulation study

**Figure 4:**
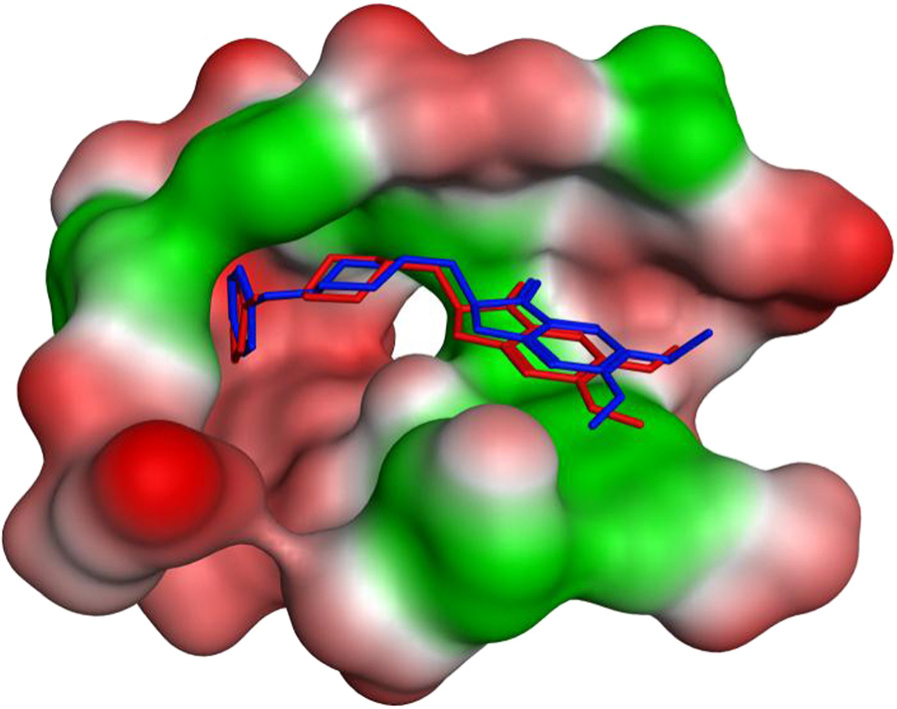
The superposition of the docked Donepezil (blue) and reference Donepezil (red) into the active site of the AChE.

In order to investigate the binding mode for interaction of the target compounds with AChE, docking studies were performed. First the co-crystallized ligand was docked back into the binding site of the enzyme and superposed with the native ligand (Figure 4).

**Figure 5:**
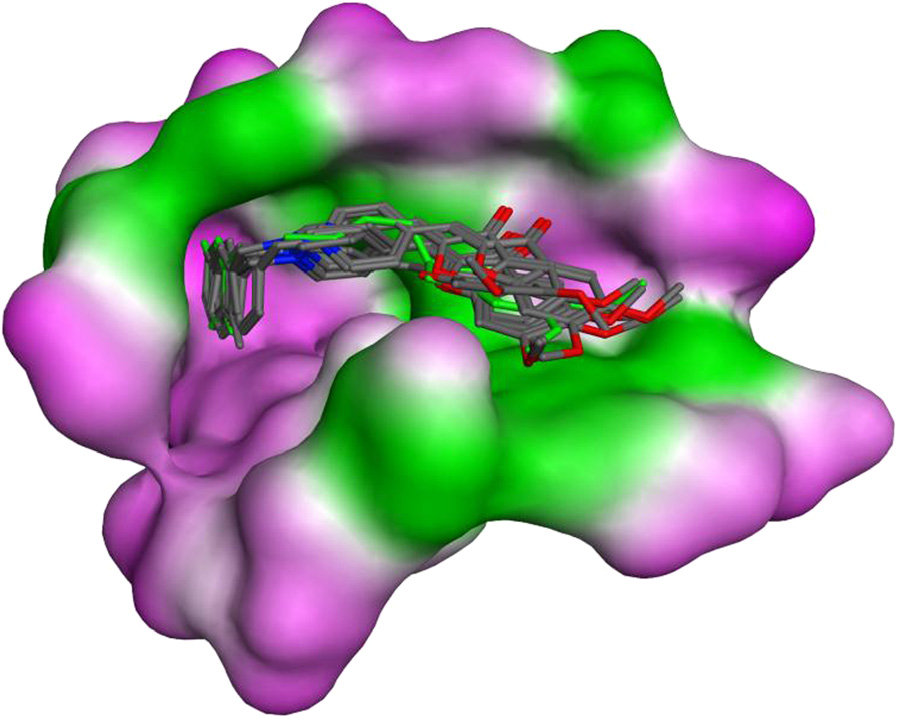
The superposition of best docked poses of all target compounds (colored by element) as well as Donepezil (green) into the gorge of AChE.

Being satisfied with the reasonable RMSD between the docked and native ligands (RMSD = 0.78 Å), the docking protocol was verified for further docking studies of the target compounds. The best docked poses of the synthesized compounds on the target were superposed and showed to be similarly docked in the gorge of AChE. As shown in Figure 5, this pattern of orientation resembled very much to that observed for donepezil.

**Figure 6:**
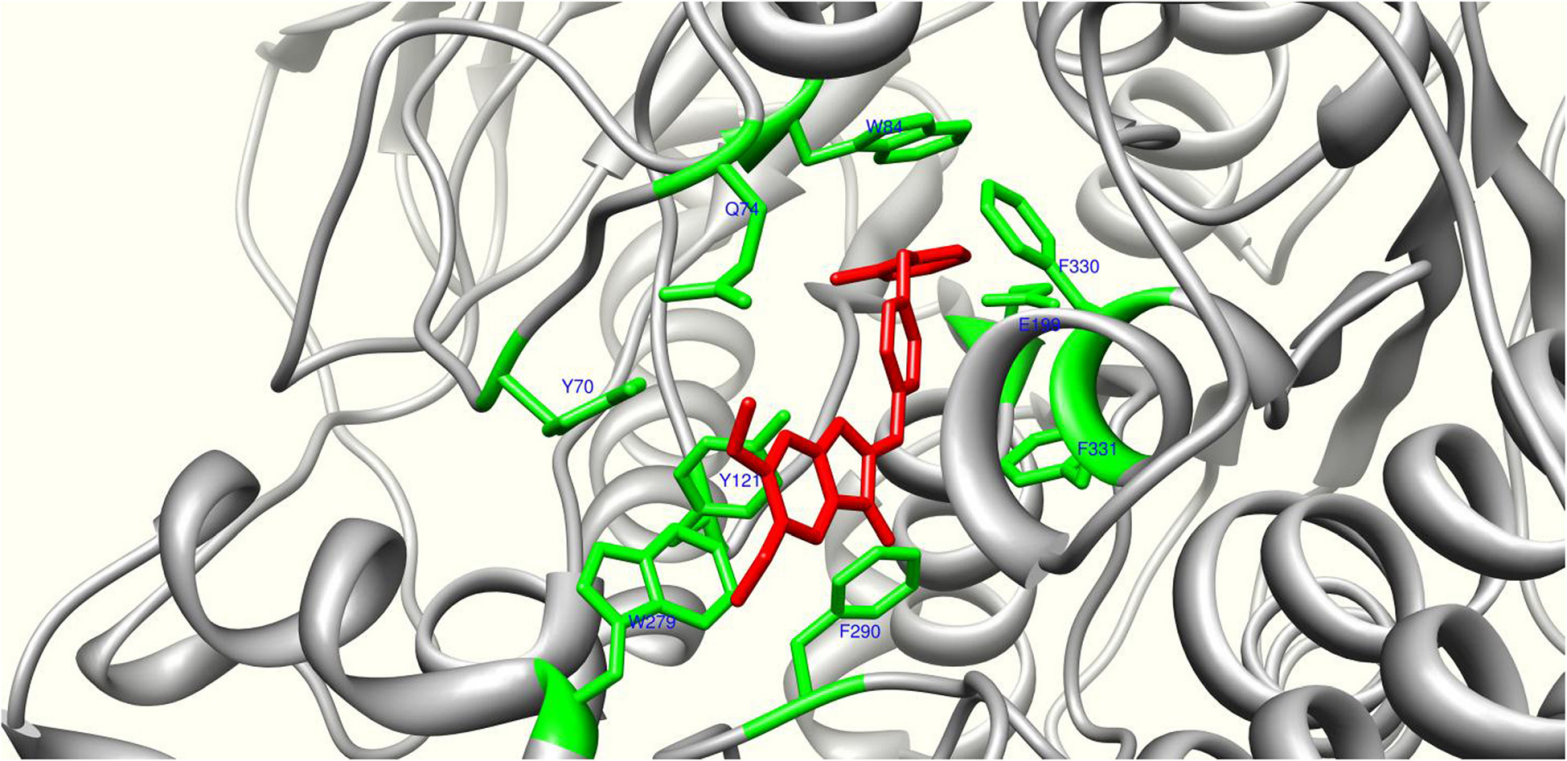
The interacting mode of the most active compound 5b with the active site of AChE.

In order to figure out the binding mode for interaction of the compounds, the most active compound (5b) was subjected to further analysis. As depicted in Figure 6, the 5,6-dimethoxybenzofuranone fragment of the ligand was well accommodated in the PAS (Peripheral Anionic Site) through a π−π stacking of the phenyl ring of benzofu-ranone and indole moiety of Trp279. This interaction was reinforced by a hydrogen bonding between the 6-methoxy of 5b and the hydroxyl group of Tyr70. In addition, the benzyl pyridinium part of the ligand was oriented towards the anionic site (AS) composed by Phe330, Trp84 and Glu199. The interactions responsible for stabilization of bezyl pyridinium in the active site included two π−π stacking and a π-cation interaction. The π-cation interaction was formed between Phe330 and the quaternary nitrogen of pyridine ring. Regarding the two π−π interactions, one was between 2-flouro substituted phenyl ring and Trp84 and the other between pyridine ring and Phe330. The same binding mode and interactions were also observed for the reference drug (Donepzil) [23].

## Conclusions

In this study, we presented new benzofuranone-based derivatives of the pyridinium type. The inhibitory activity of new synthesized compounds were tested toward AChE using slightly modified Ellman’s method and compared to those of other derivatives of this class synthesized earlier by our research group. Regarding the biological data, it was revealed that all tested compounds inhibited the AChE in nanomolar range concentration, in which compound 5b, was the most potent compound against AChE. Furthermore a same binding mode and interactions was observed for the reference drug Donepzil and compound 5b in docking study.

In conclusion, 5,6-dimethoxybenzofurane derivatives containing methylbenzyl substituent on pyridine ring demonstrated to be more potent than their corresponding 6-ethoxy and 6-propoxy benzofuranone derivatives [11].

## Competing interests

The authors declare that they have no competing interests.

## Authors’ contributions

HN participated in the synthesis of compounds and performing biological assay. AF, Ash, AA and MP contributed in design of compounds, supervision of synthetic part, elucidation of the target compounds structure and manuscript preparation. AM, AS and AV performed the docking study and participated in manuscript preparation. NH partook in synthetic section. The biological assay part is supervised by VS and MA. All authors read and approved the final manuscript.
